# Metabolic Resistance and Not Voltage-Gated Sodium Channel Gene Mutation Is Associated with Pyrethroid Resistance of *Aedes albopictus* (Skuse, 1894) from Cambodia

**DOI:** 10.3390/insects15050358

**Published:** 2024-05-15

**Authors:** Sébastien Marcombe, Bros Doeurk, Phoutmany Thammavong, Tuba Veseli, Christian Heafield, Molly-Ann Mills, Sedra Kako, Marcelly Ferreira Prado, Shakira Thomson, Saffron Millett, Timothy Hill, Imogen Kentsley, Shereena Davies, Geethika Pathiraja, Ben Daniels, Lucianna Browne, Miranda Nyamukanga, Jess Harvey, Lyranne Rubinstein, Chloe Townsend, Zack Allen, Christopher Davey-Spence, Adina Hupi, Andrew K. Jones, Sebastien Boyer

**Affiliations:** 1 Medical Entomology and Vector-borne Diseases Laboratory, Institut Pasteur du Laos, Ministry of Health, Vientiane P.O. Box 3560, Laos; sebastienmarcombe@gmail.com (S.M.); p.thammavong@pasteur.la (P.T.); 2 Vector Control Consulting—South East Asia Sole Co., Ltd., Vientiane P.O. Box 3463, Laos; 3 Medical and Veterinary Entomology Unit, Institut Pasteur du Cambodge, 5 Boulevard Monivong, Phnom Penh P.O. Box 983, Cambodia; bros@pasteur-kh.org (B.D.); sboyer@pasteur-kh.org (S.B.); 4 Department of Biological and Medical Sciences, Oxford Brookes University, Headington, Oxford OX3 0BP, UK; tuba_veseli02@hotmail.com (T.V.); christianjohnheafield@gmail.com (C.H.); molly.mills@ukhsa.gov.uk (M.-A.M.); 19186543@brookes.ac.uk (S.K.); marcelly.prado@ouh.nhs.uk (M.F.P.); shakirathomson@gmail.com (S.T.); 19056060@brookes.ac.uk (S.M.); timothy.hill1@student.lshtm.ac.uk (T.H.); imogenkentsley@icloud.com (I.K.); shereena.davies101@outlook.com (S.D.); g.pathiraja@outlook.com (G.P.); ben.daniels@syngenta.com (B.D.); lucianna2102@gmail.com (L.B.); m.nyamukanga@mft.nhs.uk (M.N.); jess.harvey@nanoporetech.com (J.H.); rubinsteinlyranne@gmail.com (L.R.); 19157654@brookes.ac.uk (C.T.); 19070375@brookes.ac.uk (Z.A.); 19149361@brookes.ac.uk (C.D.-S.); hupi.adina@gmail.com (A.H.); 5 Independent Researcher, Derby DE65 5NX, UK; 6 Independent Researcher, Oxford OX14 2RN, UK; 7 UK Health Security Agency, Porton Down, Salisbury SP4 0JG, UK; 8 Oxford University Hospitals, Churchill Hospital, Genetics Laboratories, Old Rd, Headington, Oxford OX3 7LE, UK; 9 Independent Researcher, Burnham-On-Sea TA8 1AZ, UK; 10 London School of Hygiene & Tropical Medicine, Keppel Street, London WC1E 7HT, UK; 11 Independent Researcher, Brighton BN8 4HR, UK; 12 Independent Researcher, Shrewsbury SY1 4YP, UK; 13 Independent Researcher, Wallingford OX10 7EA, UK; 14 Syngenta, Jealott’s Hill International Research Centre, Bracknell RG42 6EY, Berkshire, UK; 15 Independent Researcher, Reading RG31 4SE, UK; 16 Wythenshawe Hospital, Southmoor Rd, Wythenshawe M23 9LT, Manchester, UK; 17 Oxford Nanopore Technologies plc, Unit 3, Genesis Building, Library Avenue, Harwell, Didcot OX11 0SG, Oxfordshire, UK; 18 Independent Researcher, 69009 Lyon, France; 19 Independent Researcher, Oxford OX3 8HP, UK

**Keywords:** *Aedes albopictus*, DDT, deltamethrin, insecticide resistance, kdr mutation, malathion, permethrin, PBO, voltage-gated sodium channel

## Abstract

**Simple Summary:**

The tiger mosquito, *Aedes albopictus*, spreads the virus causing dengue fever, notably in Southeast Asian countries. To stop the spread of this disease, the *Aedes* mosquitoes are killed with chemicals known as pyrethroids. However, despite pyrethroids being highly effective insecticides, tiger mosquitoes are becoming resistant and surviving exposure to these chemicals. We have measured that tiger mosquitoes from two regions in Cambodia (the capital, Phnom Penh, and rural Pailin province) are highly resistant to pyrethroids. To determine what causes resistance, we have implicated unusually high activity of enzymes, known as P450 monooxygenases, which break down pyrethroids and thus render them harmless in the resistant mosquitoes. We have ruled out another common mechanism of pyrethroid resistance, which is changes in the mosquitoes’ DNA sequence, which prevents pyrethroids from binding to a protein known as the voltage-gated sodium channel, thereby disrupting signals being sent throughout the nervous system. Overall, our findings describe an important survey monitoring the prevalence of insecticide resistance in disease-spreading mosquitoes in Cambodia, and with the increased understanding of what causes resistance, we are in a strong position to advise that mosquito control methods alternative to pyrethroids should be implemented as soon as possible to ensure continuing management of dengue fever.

**Abstract:**

(1) Background: In Cambodia, *Aedes albopictus* is an important vector of the dengue virus. Vector control using insecticides is a major strategy implemented in managing mosquito-borne diseases. Resistance, however, threatens to undermine the use of insecticides. In this study, we present the levels of insecticide resistance of *Ae. albopictus* in Cambodia and the mechanisms involved. (2) Methods: Two *Ae. albopictus* populations were collected from the capital, Phnom Penh city, and from rural Pailin province. Adults were tested with diagnostic doses of malathion (0.8%), deltamethrin (0.03%), permethrin (0.25%), and DDT (4%) using WHO tube assays. Synergist assays using piperonyl butoxide (PBO) were implemented before the pyrethroid assays to detect the potential involvement of metabolic resistance mechanisms. Adult female mosquitoes collected from Phnom Penh and Pailin were tested for voltage-gated sodium channel (VGSC) kdr (knockdown resistance) mutations commonly found in *Aedes* sp.-resistant populations throughout Asia (S989P, V1016G, and F1534C), as well as for other mutations (V410L, L982W, A1007G, I1011M, T1520I, and D1763Y). (3) Results: The two populations showed resistance against all the insecticides tested (<90% mortality). The use of PBO (an inhibitor of P450s) strongly restored the efficacy of deltamethrin and permethrin against the two resistant populations. Sequences of regions of the *vgsc* gene showed a lack of *kdr* mutations known to be associated with pyrethroid resistance. However, four novel non-synonymous mutations (L412P/S, C983S, Q1554STOP, and R1718L) and twenty-nine synonymous mutations were detected. It remains to be determined whether these mutations contribute to pyrethroid resistance. (4) Conclusions: Pyrethroid resistance is occurring in two *Ae. albopictus* populations originating from urban and rural areas of Cambodia. The resistance is likely due to metabolic resistance specifically involving P450s monooxygenases. The levels of resistance against different insecticide classes are a cause for concern in Cambodia. Alternative tools and insecticides for controlling dengue vectors should be used to minimize disease prevalence in the country.

## 1. Introduction

The global impact of vector-borne diseases (VBDs) has been significant over the last decade, leading to over 700,000 deaths annually [1]. In tropical and subtropical regions, *Aedes* mosquitoes transmit major VBDs, such as dengue, chikungunya, and Zika [1]. *Aedes aegypti* (Goeldi, 1905) and *Ae. albopictus* (Skuse, 1894) are the main vectors [2,3,4] and they are predominant worldwide, including in Southeast Asia [1]. *Aedes albopictus* has been associated with dengue outbreaks in all continents except Antarctica [5,6,7,8,9,10,11,12].

In Cambodia, the Ministry of Health has been monitoring the incidence of dengue every year since the massive outbreak in 1995 with more than 400 deaths [13,14]. The most recent epidemics were in 2018 (9445 cases), 2019 (9298 cases), and 2023 (over 24,000 cases) [15,16]. The main vector for dengue transmission in urban areas in Cambodia is *Ae. aegypti,* while *Ae. albopictus,* originating from the forests of Southeast Asia, is more associated with dengue transmission in rural areas [17]. However, an entomological study implemented in 2019 across the capital city, Phnom Penh, highlighted a recent invasion of *Ae. albopictus* throughout the entire city [17], indicating a more important role of this species in the transmission of dengue in urban areas as observed in other countries [18,19].

With an active circulation of dengue viruses within Southeast Asia, the incidence of dengue is becoming more common in Cambodia [15]. This will continue to be an important health concern for the country, as well as neighboring countries [20]. The control of disease vectors in Cambodia relies mainly on insecticide use [21]. The organophosphate temephos (Abate^®^) has been the most broadly used insecticide in Cambodia since 1992 for targeting larval stages of *Ae. aegypti* [21], while adult *Ae. aegypti* mosquitoes are mainly targeted with pyrethroid insecticides, which have been used since the late 1980s [21]. Recent studies implemented in Cambodia showed that *Ae. aegypti* populations from rural and urban areas were considerably resistant to pyrethroid insecticides [21,22,23]. However, there are no data on the insecticide susceptibility of *Ae. albopictus* in Cambodia.

Resistance of *Ae. albopictus* to the main classes of insecticides used in public health for vector control have been recorded around the world, including Southeast Asia [4,18,20,24,25,26,27,28,29,30,31,32]. Insecticide resistance in mosquitoes is mainly associated with the over-expression of detoxification enzymes (metabolic-based resistance) and/or mutations in the sequence of the target protein that induces insensitivity to the insecticide (target-site resistance) [24]. Three large enzyme families, the cytochrome P450 monooxygenases (P450s), glutathione S-transferases (GSTs), and carboxy/cholinesterases (CCEs), have been implicated in the metabolism of insecticides [26]. The main target site mechanisms of resistance to DDT/pyrethroid insecticides involve amino acid substitutions in the voltage-gated sodium channel (VGSC), which are known as knockdown resistance (kdr) mutations [33]. The main *kdr* mutations (S989P, V1016G, F1534C, housefly *Musca domestica* numbering) responsible for DDT/pyrethroid resistance in *Ae. aegypti* have been identified worldwide [4,18], including in Southeast Asia [34]. Compared to *Ae. aegypti*, the presence of *kdr* mutations in *Ae. albopictus* has been poorly studied. However, since the first discovery of the F1534C mutation in a pyrethroid-resistant *Ae. albopictus* population from Singapore in 2011 [35], *kdr* mutation detection in *Ae. albopictus* has been implemented in other countries [36]. Mutation at the 410, 989, 1016, 1520, and 1534 amino acid positions in domains I, II, and III of the VGSC were detected in Asia in *Ae. albopictus* mosquitoes from China, Laos, Taiwan, Thailand, and Vietnam [30,36,37,38].

In this study, we investigated the levels of insecticide resistance of two *Ae. albopictus* populations from Cambodia (Phnom Penh city and Pailin province) against several insecticide families (i.e., pyrethroid, organophosphate, and DDT) using WHO bioassays. Synergist assays using piperonyl butoxide (PBO) were implemented before the pyrethroid assays to detect potential metabolic resistance mechanisms. Female mosquitoes from both populations were also analyzed for the presence of *vgsc kdr* DNA mutations.

## 2. Materials and Methods

### 2.1. Collection of Ae. albopictus Mosquito Samples

**Figure 1:**
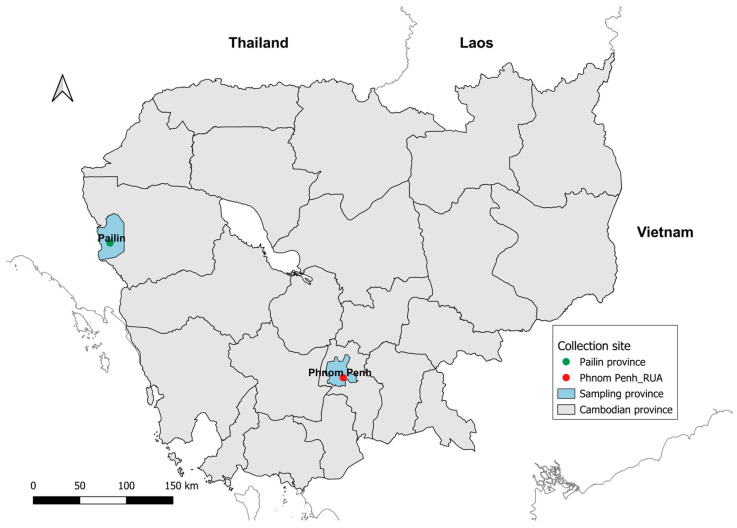
Locations of the mosquito collection sites in Cambodia, 2020.

Mosquito collections were carried out in two provinces of Cambodia (Pailin province: coordinates 12.83081, 102.6158, and Phnom Penh city, Royal University of Agriculture (RUA): 11.51196, 104.9005) (Figure 1). Mosquitoes were collected at the larval and pupal stages in Pailin using ten ovitraps without filter paper and in semi-urban areas (RUA) using a variety of sampling containers ranging from buckets, cups, fridges, tires, toilets, vases to mainly jars and plastic containers. Overall, more than 5000 larvae and pupae were collected from five different sites in Pailin during the 22nd–26th of June, 2020, and more than 5000 larvae and pupae were collected from the RUA on the 11th of June, 2020. Mosquitoes collected within a location (Pailin or RUA) on any date were pooled together and then were brought back to the laboratory at the Institut Pasteur du Cambodge and maintained under controlled conditions for rearing until adults (F1 generation) following previously described standardized techniques [21,22]. Using morphological keys to identify species [39,40], live adult *Ae. albopictus* mosquitoes were separated and kept for breeding. Then, eggs of the F1 generation were sent to the Institut Pasteur du Laos and reared to adults for the insecticide resistance bioassays. Mosquito specimens after pyrethroid insecticide tests from each population were stored in tubes with silica gel at −80 °C and sent to Oxford Brookes University laboratory for molecular analysis.

### 2.2. Insecticide Resistance Bioassays

Adult bioassays were run using filter papers treated with diagnostic doses of deltamethrin (0.03%), permethrin (0.25%), malathion (0.8%), or DDT (4%) following WHO protocols [41]. For each insecticide test, four batches of twenty-five non-blood-fed females (2–5 days old) were introduced into holding tubes and maintained for 60 min at 27 ± 2 °C and a relative humidity of 80 ± 10%. The adult mosquitoes were then transferred into insecticide exposure tubes for 60 min and then transferred back to the holding tubes. Mortality was recorded 24 h after exposure where mosquitoes were maintained in similar conditions of temperature and humidity. 

Synergist bioassays using the specific enzyme inhibitor, piperonyl butoxide (PBO), were conducted to address the potential role of P450s in insecticide resistance. Adult mosquitoes were exposed to a sub-lethal concentration of PBO (4%) for 1 h prior to treatment with deltamethrin or permethrin following the same WHO protocol for adult bioassays [41].

Following WHO criteria, a population was resistant if the mortality rate was below 90%. Resistance was suspected when the mortality rate ranged from 90% to 98%, and a population was considered susceptible when the mortality rate was over 98% [41]. A susceptible United States Department of Agriculture (USDA) strain of *Ae. aegypti* was used as the control to test the effectiveness of the insecticides [42].

### 2.3. Detection of Mutations in the Voltage-Gated Sodium Channel

Genomic DNA was extracted from individual adult *Ae. albopictus* females using 250 μL Trizol (Fisher Scientific, Loughborough, UK) following the manufacturer’s protocol. With 2 μL of extracted DNA as a template, the polymerase chain reaction (PCR) using the Q5^®^ High-Fidelity Kit (New England Biolabs, Ipswich, MA, USA) was used to amplify four sections of the *vgsc* gene where mutations giving rise to pyrethroid resistance are known to occur [43]. Thus, TM6 in domain I (amino acids 369–451, *Musca domestica* numbering) was amplified by the primers 5′-ACTAACCAAAACTCCCACTAC-3′ (forward) and 5′-GAAAAACAAAGAGATTTAAGAGAG-3′ (reverse), with resulting amplification products being sequenced with the 5′-TCTTCGTGGTGTGCAAAACAG-3′ (forward) oligonucleotide. TM6 in domain II (amino acids 952-1052 including intron 20) was amplified by 5′-CAGACAATGTGGATCGCTTC-3′ (forward) and 5′-GATATCCGGTTGAACGCCTC-3′ (reverse) and then sequenced using 5′-CGGACAAGGACCTGCCAC-3′ (forward). TM6 in domain III (amino acids 1446-1593 including intron 29) was amplified with either 5′-TGCGTCGACAAGAACAAGAC-3′ (forward), 5′-AAGACGACGCTGTCGCAC-3′ (forward), or 5′-AGAACTACACGTGGGAGAAC-3′ (forward) and 5′-CCTAGGCCGAGGGATAGC-3′ (reverse) and then sequenced using 5′-TTCAGCGGCTTCTTCGAGC-3′ (reverse). TM6 in domain IV (amino acids 1692-1780) was amplified by 5′-TCGAGAAGTACTTCGTGTCG-3′ (forward) and 5′-CAGCGATGTACATGTTGATAAC-3′ (reverse) and then sequenced with 5′-CCACGTTGCTCCGAGTCG-3′ (forward). The PCR products were purified using the SmartPure PCR Kit (Eurogentec, Seraing, Belgium) before being sequenced at SourceBioscience (https://genomics.sourcebioscience.com/ accessed on 29 November 2021). Sequence chromatograms were visualized using Chromas (available online at https://technelysium.com.au/wp/chromas/ accessed on 6 December 2021) and compared to the *Ae. albopictus* sequence with Accession No. XM_029865086.1.

### 2.4. Sequence Analysis and Accession Numbers

Sequence alignments were constructed using Clustal X2 [44] using default settings and displayed using Genedoc (http://nrbsc.org/gfx/genedoc/index.html accessed on 15 February 2024). 

Novel sequences of intron variants have been deposited in the National Center for Biotechnology Information (https://www.ncbi.nlm.nih.gov/WebSub/ accessed on 4 March 2024) and have accession numbers of PP426602 (intron20Var74), PP426603 (intron20Var84b), PP426604 (intron20Var85), PP426605 (intron20Var89a), PP426606 (intron20Var89b), PP426607 (intron20Var90b), PP426608 (intron20Var91), PP426609 (intron20Var94a), PP426610 (intron20Var94b), PP426611 (intron29Var67), PP426612 (intron29Var82), and PP426613 (intron29Var83d).

## 3. Results

### 3.1. Bioassays

**Figure 2:**
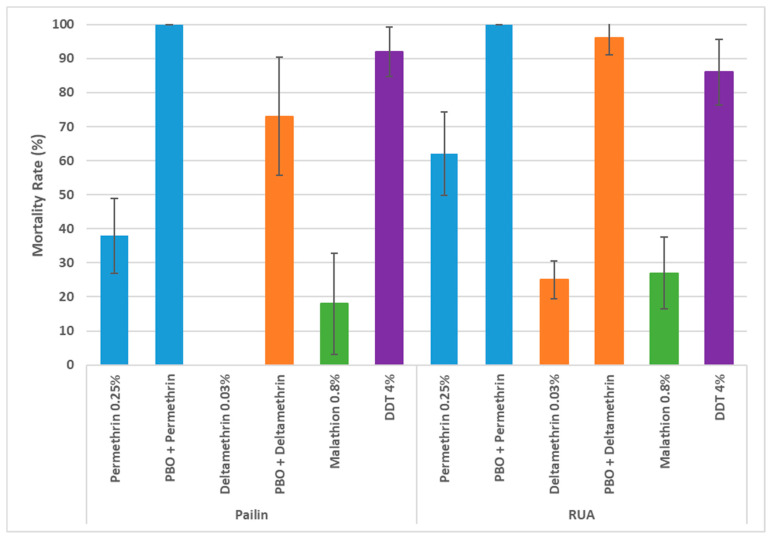
Mortality (%) of adult *Aedes albopictus* from Pailin and Phnom Penh city (Royal University of Agriculture (RUA)) after exposure to the selected insecticides and the inhibitor PBO (4%).

For all bioassays, mortality in the control tubes, including the susceptible *Ae. aegypti* strain USDA with insecticides for the positive control and both Cambodian *Ae. albopictus* populations without insecticide for the negative control, never exceeded 5%, so no mortality correction was necessary. The two *Ae. albopictus* populations tested were resistant against all the insecticides tested (mortality < 90%), except for the population from Pailin, which showed 92% mortality with DDT, indicating possible resistance (Figure 2). Thus, the populations from Phnom Penh (RUA) and Pailin province showed, respectively, 27% and 18% mortality with malathion, 25% and 0% with deltamethrin, and 62% and 38% with permethrin. However, when exposed to PBO prior to the pyrethroid tests, the mortality of mosquitoes from both populations significantly increased. In Phnom Penh, mortality to deltamethrin increased from 25% to 96%, and permethrin increased from 62% to 100%. In Pailin, mortality to deltamethrin increased from 0% to 73% and 38% to 100% for permethrin.

### 3.2. Detection of Mutations and Intron Variants in the Voltage-Gated Sodium Channel

**Figure 3:**
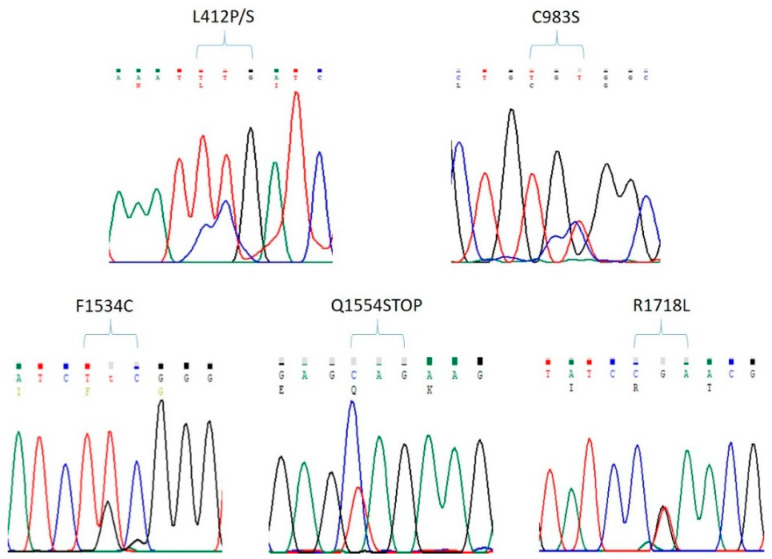
Example of DNA sequence chromatograms showing the heterozygous non-synonymous mutations in the *vgsc* gene of *Ae. albopictus* identified in this study.

Genomic DNA encoding for TM6 in domains II and III of the *vgsc* gene from individual female *Ae. albopictus* mosquitoes sampled in Phnom Penh or for TM6 in domains I-IV from mosquitoes taken from Pailin were amplified and analyzed for mutations associated with pyrethroid resistance [43]. Sequence chromatograms showed no known pyrethroid resistance mutations in domains I, II, and IV (Table 1), whilst only one of the eighty-one mosquitoes from Phnom Penh was heterozygous for the F1534C mutation (TTC to TGC) (Table 1, Figure 3). Also, other mutations (S1000Y and I1532T) reported for *Ae. albopictus* from China [45] were not found here. 

Four novel non-synonymous mutations were detected in mosquitoes collected from Pailin (Figure 3), where three of the seventy-eight domain I sequences (mutation frequency 1.92%) analyzed were heterozygous for either L412S (TTG to TCG) or L412P (TTG to CCG), whilst a different three mosquitoes were heterozygous for C983S (TGC to either TCC or TCT) located in domain II (mutation frequency 2.05%). Another two mosquitoes were heterozygous for Q1554STOP (CAG to TAG) in domain III (mutation frequency 1.85%), and a further five mosquitoes were heterozygous for R1718L (CGA to CTA) in domain IV (mutation frequency 3.16%). Synonymous mutations located in all four domains were also observed in more than one mosquito as follows: domain I L412L (TTG to CTG); domain II V981V (GTG to GTA), C983C (TGC to TGT), S989S (TCC to TCT), C993C (TGC to TGT); domain III D1505D (GAC to GAT), G1513G (GGA to GGC), P1516P (CCG to CCA), Y1527Y (TAC to TAT), F1528F (TTT to TTC), F1531F (TTC to TTT), F1534F (TTC to TTT), F1543F (TTC to TTT), G1545G (GGT to GGC), I1547I (ATC to ATT), I1548I (ATC to ATT), D1549D (GAC to GAT), N1550N (AAC to AAT), K1556K (AAG to AAA); and domain IV F1722F (TTT to TTC), L1738L (CTG to TTG), G1749G (GGG to GGC), F1753F (TTC to TTT), H1755H (CAC to CAT), V1756V (GTG to GTA), S1760S (AGC to AGT), N1767N (AAT to AAC), F1768F (TTT to TTC), and S1774S (AGT to AGC).

**Figure 4:**
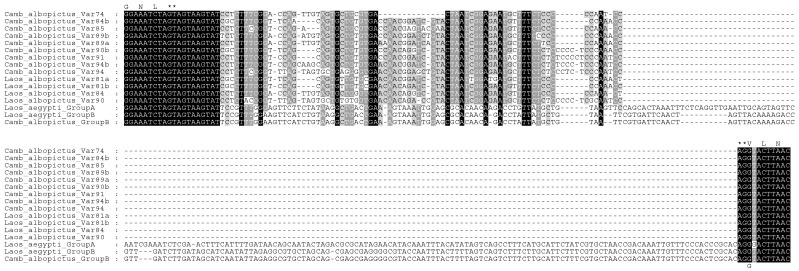
Alignment of the variable intron 20 sequences and parts of flanking exons found in the *vgsc* gene of *Aedes albopictus* collected in Laos [37] and Cambodia (current study). Group A and B intron sequences characterized in *Aedes aegypti* [46,47,48,49] are included here for comparison with the group B sequence identified in *Ae. albopictus*. Black and gray shading indicates the degree of conservation. Amino acid residues corresponding to coding regions are shown at the top of the alignment, whilst the mutated amino acid (V1016G) found in group A is shown at the bottom. Splice donor and acceptor sites are marked by asterisks.

Ten different sequences were observed for intron 20 (Figure 4). Nine of these intron sequences varied in size from 74 to 94 nucleotides, consisting of 74, 84, 85, 89, 89, 90, 91, 94, and 94 bp. They have been, respectively, denoted here as intron20Var74 (Accession No. PP426602) and intron20Var84b (PP426603) to differentiate it from intron20Var84 from Laos [37], intron20Var85 (PP426604), intron20Var89a (PP426605), intron20Var89b (PP426606), intron20Var90b (PP426607), intron20Var91 (PP426608), intron20Var94a (PP426609), and intron20Var94b (PP426610). The tenth intron 20 sequence consists of 237 bp and is identical to the previously characterized intron B variant [46] found in *Ae. aegypti* from Laos [37]. None of the intron variants observed here were identical to any of the four variants seen in *Ae. albopictus* collected from Laos [37].

**Figure 5:**
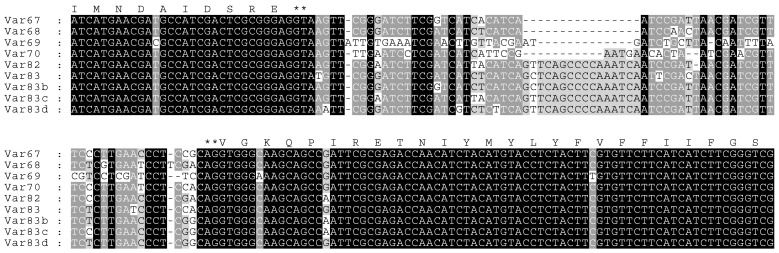
Alignment of the variable intron 29 sequences and parts of flanking exons found in the *vgsc* gene of *Aedes albopictus* collected in Laos [37] and Cambodia (current study). Black and gray shading indicates the degree of conservation. Amino acid residues corresponding to coding regions are shown at the top of the alignment. Splice donor and acceptor sites are marked by asterisks.

Seven different sequences were observed for intron 29 (Figure 5). Four of these sequences, consisting of 68, 70, 83, and 83 bp, were respectively identical to intron29Var68, intron29Var70, intron29Var83, and intron29Var83b detected in *Ae. albopictus* from Laos [37]. The remaining three sequences consist of 67, 82, and 83 bp and thus are denoted here as intron29Var67 (PP426611), intron29Var82 (PP426612), and intron29Var83d (PP426613), respectively. Intron29Var82 contained the CCA codon for the synonymous mutation at P1516P [37], whilst the codon was CCG in intron29Var67 and intron29Var83d (Figure 5).

## 4. Discussion

In this paper, we demonstrate that two different populations of *Ae. albopictus* from Cambodia were resistant to pyrethroid insecticides, highlighting the need for alternative insecticides and/or strategies for controlling this vector. The use of PBO strongly restored the efficacy of deltamethrin and permethrin against both populations, indicating the involvement of specific detoxification enzymes, the P450s monooxygenases. Metabolic resistance, including P450s, is a common occurrence in mosquitoes and has been reported against insecticides used in public health and agriculture [4]. Fewer studies have shown the involvement of P450s in *Ae. albopictus* from Asia that are resistant to pyrethroids. In Malaysia, permethrin insensitivity was mediated by metabolic resistance through the over-expression of P450 genes (CYP6 family) in *kdr*-free *Ae. albopictus* populations [50]. Also, in China, Zou et al. [51] reported the important role of three CYP6 genes in deltamethrin resistance in several *Ae. albopictus* populations. To further understand metabolic resistance in *kdr*-free *Ae. albopictus* populations from Cambodia, as well as neighboring countries, it is necessary to characterize the involvement of P450 genes (upregulation/gene copy number variations [CNVs]) [52]. It is also important to conduct similar studies on the populations of *Ae. albopictus* in Cambodia that are resistant to malathion, as CNVs of carboxylesterase genes were found to be responsible for organophosphate resistance in *Ae. aegypti* populations from Southeast Asia [34,53].

We also report here the first survey for pyrethroid resistance mutations in the *vgsc* gene in *Ae. albopictus* from Cambodia. Genomic DNA of the *vgsc* gene encoding for transmembrane region 6 in each of the four domains, where mutations giving rise to pyrethroid resistance are known to occur [43], were sequenced. Only one mosquito was found to have a mutation, which was heterozygous for F1534C (Figure 3 and Table 1). This lack of *kdr* mutations highlights the involvement of P450s as being a predominant mechanism for pyrethroid resistance in Cambodia. Sequencing PCR products resulted in the identification of novel non-synonymous mutations in the vicinity of recognized pyrethroid-resistance mutations. Thus, L412P/S is close to V410L in domain I, C983S is close to S989P in domain II, and R1718L is located in domain IV with D1763Y. Intriguingly, a fourth mutation was identified that introduced a stop codon at position 1554 in domain III, which would result in a truncated protein that is unlikely to be functional. Truncated *vgsc* variants arising from in-frame stop codons have been reported before, for example, in the mosquito *Culex pipiens* [54]. The *Ae. albopictus* mosquitoes observed here were heterozygous for the wild type and Q1554STOP, which may result in abnormal neuronal signaling, as indicated by mice models with heterozygous loss of function for the VGSC, SCN2A [55]. Many more synonymous mutations located in all four domains were detected, including at sites associated with pyrethroid resistance (S989 and F1534). The synonymous mutation at F1534 has also been observed in *Ae. albopictus* from China, Hawaii, and Los Angeles [30]. Other synonymous mutations observed here, such as C983C, C993C, and P1516P, have been detected in *Ae. albopictus* from several different countries, including Hawaii, Italy, Japan, and Singapore [56]. Since differential codon use for the same amino acid may affect protein expression, conformation, and function [57], it has been suggested that it may be prudent to consider synonymous mutations in future studies of insecticide resistance [36]. It remains to be determined whether the synonymous and non-synonymous mutations identified here contribute to pyrethroid resistance.

We identified a variant in intron 20 in the *vgsc* gene of *Ae. albopictus*, which is similar to type B found in *Ae. aegypti* (Figure 4) [49]. Previous studies have found that another intron variant in *Ae. aegypti*, type A, is linked to the presence of resistance mutations V1016G and S989P [37,46,47]. Our finding of the type B intron, therefore, is in accord with the absence of these mutations. The majority of intron 20 variants observed, however, are considerably shorter than the type B intron and, so far, appear to be found only in *Ae. albopictus* and not *Ae. aegypti*. These shorter variants found in *Ae. albopictus* from Cambodia all differ in sequence from those identified in mosquitoes collected in Laos [37].

Multiple variants for intron 29 were also detected (Figure 5). Four of these (intron29Var68, intron29Var70, intron29Var83, and intron29Var83b) have identical sequences to those found in *Ae. albopictus* from Laos [37], whilst the remaining three are novel. All of the intron 29 sequences obtained from *Ae. albopictus* from Laos and Cambodia are different from those sequenced from *Ae. albopictus* collected in Shanghai, China [58]. It was found that in the mosquitoes from Shanghai, intron 29 consisting of 68 bp (denoted intron B) had a significant tendency to be associated with non-synonymous mutations at 1532 and 1534. It will be of interest to see whether introns with similar sequences are associated with pyrethroid resistance mutations in *Ae. albopictus* from other countries, thereby pointing to particular intron 29 variants playing a role in the regulation of gene mutation.

## 5. Conclusions

Constant monitoring programs for insecticide resistance levels and mechanisms should be implemented in Cambodia, where vector-borne diseases have an important public health impact. Even in the absence of *kdr* mutations in the two *Ae. albopictus* populations from Cambodia, it has been shown that several mutations in the *vgsc* genes are responsible for high pyrethroid resistance in *Ae. aegypti* populations in the country [23]. Thus, a careful picture of the diffusion of *kdr* mutations due to constant insecticide pressure represents an important milestone in the implementation of vector control plans and the triggering of novel research on alternative strategies for mosquito-borne infections. Also, the development of molecular tools to track metabolic resistance alleles and the comprehensive understanding of the genetic bases of metabolic resistance could represent significant steps forward for public health vector control.

## Figures and Tables

**Table 1:** Frequencies of pyrethroid-resistant mutations [43] in the four domains (Dom) of the *vgsc* gene of *Aedes albopictus* collected from Pailin and Phnom Penh city, Royal University of Agriculture (RUA). No. is the number of mosquitoes analyzed. —denotes a value that was not determined.

		Dom I	Dom II					Dom III			Dom IV
	Mutation Allele	V410L VV	L982W LL	S989P SS	A1007G AA	I1011M II	V1016G VV	T1520I TT	F1534C FF	FC	D1763Y DD
RUA	No.	–	16	16	16	16	107	81	80	1	–
	Total	–	16	16	16	16	107	81	81		–
	Mutation frequency (%)	–	0	0	0	0	0	0	0.62		–
Pailin	No.	78	73	73	73	73	60	54	54	0	79
	Total	78	73	73	73	73	60	54	54		79
	Mutation frequency (%)	0	0	0	0	0	0	0	0		0

## Data Availability

The raw data supporting the conclusions of this article will be made available by the authors upon request.
